# Engineering Permissive Insertion Sites in the Bacteriophage Phi29 DNA-Linked Terminal Protein

**DOI:** 10.1371/journal.pone.0164901

**Published:** 2016-10-25

**Authors:** Pablo Gella, Margarita Salas, Mario Mencía

**Affiliations:** Centro de Biología Molecular “Severo Ochoa” (Consejo Superior de Investigaciones Científicas–Universidad Autónoma de Madrid), Universidad Autónoma de Madrid, Cantoblanco, 28049 Madrid, Spain; Tulane University Health Sciences Center, UNITED STATES

## Abstract

Many different DNA delivery vehicles have been developed and tested, all with their advantages and disadvantages. The bacteriophage phi29 terminal protein (TP) is covalently linked to the 5’ ends of the phage genome during the DNA replication process. Our approach is to utilize this TP as a platform to incorporate different protein or peptide modules that can target the DNA to the interior of the cell, to the nucleus, or even to subcellular compartments. In order to be able to insert different peptide modules on the TP sequence to endow it with desired functions and/or eliminate unwanted regions of the protein, we have carried out a transposition screening to detect insertion-permissive points on the sequence of the TP. We report the functional characterization of 12 insertion mutants of the TP, and the identification of one site at position 38 that allows the insertion of peptides up to 17 amino acids in length while maintaining the ability of the TP to support DNA amplification *in vitro*. A protein with one insertion at that position containing a cysteine residue, a linker, and a thrombin recognition site was purified and its amplification activity was optimized.

## Introduction

DNA delivery into eukaryotic cells is a task that ranges from trivial to extremely complicated depending on the exact outcome intended. In order to achieve *in vivo* efficiency, quantitative yield, or subcellular compartment targeting, a large number of approaches have been tested [1]. In general, these delivery methods can be subdivided in two: viral vectors and non-viral protocols [2, 3]. The viral vectors enjoy high efficiency and in some cases can be targeted to specific cell types, however, the method is complex, expensive, and concerns about safety remain. Non-viral vectors, on the other hand, are easier to use but are much less efficient and the targeting is difficult. There is effectively no system that enjoys the advantages of the two methods while not being affected by their inconvenients.

In an effort to produce a DNA delivery vehicle that could have the beneficial characteristics of the two afore mentioned types of methods, we have focused on the engineering of a well-studied model, the replication system of bacteriophage phi29 (see [4] for a review), that could constitute the basis for a new versatile method for gene delivery.

The genome of bacteriophage phi29 is a 19 Kb double-stranded DNA that has a terminal protein (TP) covalently linked at each 5´ end [5]. The replication of the phage genome is carried out by four essential proteins encoded by the phage. These proteins can be purified to reconstitute an efficient replication-amplification reaction *in vitro* that simulates the replication of the TP-DNA of the phage *in vivo* [6]. These four proteins are, the DNA polymerase, the TP, the origin-recognition double-stranded DNA binding protein, p6, and the phage SSB, p5. At the initiation of the phi29 genome replication, a TP molecule becomes covalently linked to each of the 5´ends of the newly synthesized genome where they remain for the whole viral cycle [7]. Apart from acting as the primer for replication, the presence of the TP linked at the 5’ ends (named parental TP) probably protects these ends from degradation inside the cell and the TP has also been shown to strengthen the *in vitro* replication reaction by recruiting the replicative complexes to the end-origins [8]. It is also worth mentioning that the phi29 DNA polymerase, working alone, is a very useful enzyme for molecular biology as it is the basis of widely used whole genome amplification methods (as the multiple displacement amplification) due to its great processivity, strand displacement capacity and fidelity [9]. Focusing on the phage genome replication, we have recently developed an amplification method based on the four replication proteins of phi29 that allows the TP-primed amplification of linear heterologous DNAs [10]. The heterologous DNAs amplified are attached to the 190 bp DNA right and left ends of the phi29 genome that act as efficient origins for this TP-primed amplification. These ends can be appended to the heterologous DNAs to be amplified by cloning or ligation. The end product of this type of amplification is a given DNA with the ends of phi29 and the TP covalently linked at both 5´ends [10].

The phi29 TP has been shown to perform several different functions needed to orchestrate the dynamics of the phage genome. Structurally, the TP has three domains [11]. The carboxy-terminal domain (Ct domain, amino acids 173–266) docks to the dsDNA binding cleft of the DNA polymerase, mimicking the shape and surface charge of dsDNA, to work as a primer for the initiation of the replication. So, the first nucleotide, dAMP, is esterified to the hydroxyl group of serine 232 on the TP [12]. The TP intermediate domain (amino acids 74 to 172) provides most of the surface of interaction with the DNA polymerase outside the DNA binding cleft, and it is thought to be instrumental in the conformational changes that occur when the TP is released from the polymerase. And, lastly, the amino-terminal domain (Nt domain, amino acids 1 to 73), whose structure was not resolved in the crystal, has been shown to bind dsDNA in a non-sequence dependent manner [13,14]. Also, this domain appears to be important for efficient replication of the phage genome *in vivo* [14], although it is partially dispensable for *in vitro* replication [15]. *In vivo*, the TP, through its N-terminal domain, has been reported to localize in the bacterial nucleoid and to direct the DNA polymerase and the phage genome to the nucleoid [14,16], where the transcription and first stages of DNA replication take place. So, according to our current model the TP linked to the phage DNA acts as a nucleoid targeting signal that promotes localization in the nucleoid for the whole genome. Finally, the three domains of the TP are connected by two non-rigid linkers that allow the proposed inter-domain movements required for the proper dynamics of the TP in the early replication process [11].

In a somewhat unexpected finding we have shown that the phi29 TP, when expressed in mammalian cells, efficiently localizes in the nucleus and we were able to show that TP amino acids 14 to 38 constitute a *bona fide* eukaryotic nuclear localization signal (NLS) [17]. This NLS, curiously, is within the DNA binding domain of the TP, and the amino acids 14 to 38 are essential for that binding. Furthermore, we showed that the transfection of mammalian cells with DNAs with TP covalently bound improved the transfection efficiency when compared with the transfection of an identical linear DNA without TP [17]. The previous work suggests that the DNA-bound TP could work as a targeting module that could ferry the bound DNA to the mammalian cell nucleus and perhaps to other cell compartments.

Our approach consists on utilizing the TP as a platform to incorporate different protein or peptide modules that can perform functions that target the DNA to the interior of the cell, to the nucleus or even to other subcellular compartments, such as mitochondria. For this purpose, we considered very useful to explore the structural permissivity to insertions of the TP, because it would give the possibility to insert different peptide modules on its sequence so we can endow it with desired functions and/or eliminate unwanted regions. The modules to be added to permissive sites on the TP could be TAT or other cell-penetrating peptides [18], protease recognition sites to trim the TP, or even mitochondrial targeting peptides.

In this sense it is useful to have in mind that Adenovirus has been a common vehicle to deliver genes to be expressed in eukaryotic cells [19]. Adenovirus has, like phi29, a TP-linked linear genome [20], but, in the case of phi29, there is an efficient system available for the *in vitro* production of TP-linked linear DNA [10].

We decided to perform a transposon-based random screening because it would give us an unbiassed answer on which insertions could appear, in principle, at any point in the TP sequence. To random-scan the TP sequence for permissive sites we utilized a reported system based on the insertion, using a Tn5-based transposon, of the GFP ORF, devoid of transcription and translation initiation signals, on the target sequence [21]. In most of the insertion mutants we have isolated, the GFP insertion abrogated the TP replication activity and, subsequently, the GFP sequence in the different insertion mutants was reduced to a 15 amino acid insertion at the same place as it had been the GFP. A representative collection of TP insertion mutants was assayed for a series of activities comparing them to the wild-type protein. The insertion of the most adequate candidate (i38) was substituted with a designed sequence containing a cysteine plus a protease site, and the conditions for TP-amplification were optimized for this mutant.

## Materials and Methods

### Nucleotides and DNAs

Unlabeled nucleotides were purchased from General Electric Healthcare. [α-^32^P]dATP was supplied by PerkinElmer Inc. Oligonucleotides were obtained from Sigma-Aldrich. TP-containing phi29 DNA (TP-DNA) was prepared as described [7]. Plasmid pGA-BCL1 harboring the transposon mTn5[GFP-Nla1] (S1 File) was a kind gift from Dr. Víctor de Lorenzo [21]. Plasmid pETORPHI construction is described in [10]. The 216-bp DNA fragment corresponding to the *B*. *subtilis yshC* gene was obtained by PCR as described in [16].

### Proteins and peptides

DNA polymerase [22], TP and protein p6 [10], and protein p5 [23] were purified as described. TP insertion mutants were expressed in *E*.*coli* BL21(DE3) cells cultured in 200 ml with autoinduction medium ZYM5052 [24] overnight at 30°C and purified with the Strep-tag-Strep-Tactin purification kit (IBA) following the manufacturer instructions. Purified proteins were analyzed and quantified by SDS-PAGE and Coomassie blue staining. The quantification was performed by gel densitometry of the bands, and calculation of the protein concentration by comparison with known amounts of wild-type TP and appropriate size markers.

#### Transposition assay

The transposon was amplified using the primers Tn5Fw and Tn5Rev (see Table 1) and the plasmid pGA-BCL1 as template. The PCR product was digested with DpnI for 4 h at 37°C to eliminate the DNA input and purified using the QIAquick PCR purification kit (QIAGEN). To generate the GFP insertion library within the phage phi29 TP sequence we used the amplified transposon and the plasmid pStrepTP (S2 File) which contains the TP gene under a T7 promoter with a double Strep-tag at the N-terminal end, cloned in the vector pT7-7 [25]. The hyperactive Tn5 transposase was prepared as described [26]. The transposition reaction contained, in 10 μl, 50 mM Tris-acetate, pH 7.5, 0.15 M potassium acetate, 4 mM espermidine, 1 mM EDTA, 10 mM MgCl_2_, 0.1 μM of transposase, 0.01 μM of the amplified transposon (ratio transposase:transposon, 1:10) and 0.075 μM of plasmid. The mixture was incubated for 2 h at 37°C and was stopped by incubation for 10 min at 70°C. 1 μl aliquots were transformed into *E*.*coli* XL-1 Blue cells and selected by the kanamycin plus ampicillin double resistance. 6000 resistant clones were pooled and the plasmidic DNA was isolated by the Wizard Plus SV Miniprep purification kit (Promega). To select the transposition events that occurred within the TP gene, the plasmid library was digested with EcoRI and BamHI, and the DNA band with a molecular weight corresponding to the TP gene plus the transposon was excised from an agarose gel, purified and recloned into the pT7-7 vector digested with the same enzymes. The new library was then digested with NotI and religated to eliminate the kanamycin resistance gene, obtaining the final transposition library. This library was transformed into *E*.*coli* BL21(DE3) cells and the colonies were selected by ampicillin resistance.

**Table 1:** Oligonucleotides used in this study.

Name	Sequence 5’-3’
**Tn5Fw**	CGTCAAGGCCGCATGGTACCCA
**Tn5Rev**	CCCATGAGGCCCAGGAGCTCAG
**TransDel1**	GTTTAAACAGACGGCCGTAAGAGACAG
**TransDel2**	GAACTATACAAAGCGGCCGCAGAT

To eliminate the GFP coding sequence from the selected transposition mutants the plasmid was amplified by PCR using oligonucleotides TransDel1 and TransDel2 (see Table 1), and the DNA present in the reaction was digested with DpnI to eliminate the parental plasmid. The PCR fragment comprising the pStrepTP plasmid plus insertions of 45 bp was cut with EagI, ligated and transformed.

#### Liquid culture fluorimetry

Selected bacterial colonies were allowed to grow in 200 μl LB + ampicillin overnight at 37°C in 500 μl 96-well plates (Deepwell plates, Eppendorf) with shaking. The following day, protein expression was induced by adding 0.5 mM IPTG for 2 h. A 50 μl sample of each culture was transferred to new 96-well black plates (Greiner Bio-One), and fluorescence emission was measured in the 485 nm excitation/510 nm emission spectra, using an OPTIMA plate reader (BMG LABTECH). In each plate, 8 wells had cells containing the empty plasmid pT7-3, as a negative control (not fluorescent), and another 8 wells had cells containing the plasmid pEYFP-TP, which expresses the fusion protein YFP-TP (YFP protein fused to the N-terminal end of the TP), and they were used as a positive control (fluorescent). Fluorescence from the selected colonies was measured and its value was normalized by substracting the average of the negative control points and referring it to the positive controls as 100%.

#### GFP expression analysis by Western Blot

Colonies that produced a value of 60% or more in the fluorescence assay were tested by Western Blot to check if they contained GFP-TP fusions. 56 selected bacterial cultures bearing the TP-transposon and positive in fluorescence, were allowed to grow overnight at 37°C in 1 mL LB + ampicillin medium. The following day these cultures were diluted 1:100 in fresh LB + ampicillin and growth to exponential phase. At an OD_600_ of 0.4 GFP expression was induced by adding 0.15 mM IPTG to the medium. 1 ml aliquots were centrifuged at 14000xg for 10 min. The supernatant was removed and the pellet was resuspended in 250 μl of protein loading buffer (37 mM Tris-HCl, pH 6.8, 2% SDS, 4% (v/v) 2-mercaptoethanol and 13% (v/v) glycerol) and sonicated (6 cycles of 2 sec at 40 microns). Samples were heated for 5 min at 95°C and analyzed by SDS-PAGE in 12% acrylamide gels. Proteins were transferred to an Immobilon-P membrane (Millipore) and incubated with a primary polyclonal antibody anti-TP from rabbit (dilution 1/3000), overnight at 4°C. Antigen-antibody complexes were washed and incubated with horseradish peroxidase-conjugated antibody anti-rabbit (GE Healthcare) for 1 h at room temperature and detected with ECL (Enhanced ChemiLuminiscence) detection kit (Amersham) by autoradiography.

#### Initiation assay (TP-dAMP complex formation)

To detect the TP-dAMP complex formation in bacterial extracts, *E*.*coli* BL21(DE3) colonies expressing the selected GFP-fused TPs were allowed to grow overnight at 37°C. The following day these cultures were diluted 1:100 in fresh LB + ampicillin and they were allowed to grow to exponential phase. At an OD_600_ of 0.4, GFP expression was induced by adding 0.15 mM IPTG to the medium. 1.5 ml aliquots were centrifuged at 14000xg for 10 min. The supernatant was removed and the pellet was resuspended in 300 μl of buffer A (50 mM Tris-HCl, pH 8, 0.5 M NaCl, 1 mM EDTA, 7 mM 2-mercaptoethanol, 5% (v/v) glycerol and 0.1% (v/v) Tween-20). Cell suspensions were sonicated as indicated above. 1.5 μl aliquots of the bacterial extracts obtained were added to an incubation mixture that contained, in 25 μl, 50 mM Tris-HCl, pH 7.5, 1 mM MnCl_2_, 4% (w/v) glycerol, 1 mM DTT, 0.1 mg/ml BSA, 20 mM (NH_4_)_2_SO_4_ plus 5 μM dATP and 1 μCi [α-^32^P]dATP (ratio [α-^32^P]dATP:dATP = 1:373), 35 nM DNA polymerase and 0.73 nM phi29 TP-DNA, and was incubated for 1 h at 30°C. The reactions were stopped by adding 10 mM EDTA and 0.1% SDS, final concentration, and filtered through Sephadex G-50 spin columns. Samples were analyzed in 12% SDS-PAGE and detected by autoradiography.

To measure initiation in the presence of purified proteins, the incubation mixture contained, in 25 μl, 50 mM Tris–HCl, pH 7.5, 10 mM MgCl_2_, 5% (v/v) glycerol, 1 mM DTT, 0.1 mg/ml BSA, 20 mM (NH_4_)_2_SO_4_, 0.1 μM and 1 μCi of dATP and [α-^32^P] dATP, respectively, 150 nM of the purified wild-type TP or mutants, 30 nM of phi29 DNA polymerase and 0.63 nM TP-DNA. The mixture was incubated for 5 min at 30°C. The reactions were stopped by adding 10 mM EDTA and 0.1% SDS, final concentration. Then, the samples were filtered through Sephadex G-50 spin columns in the presence of 0.1% SDS and were analyzed by 12% SDS-PAGE and detected by autoradiography.

#### Assay of competition between TPs

The reaction was performed as for the initiation assay but in this case, 75 nM of each competitor TP were incubated together for 10 min in ice ([TP_total_] = 150 nM). The mixture of TPs was incubated with 30 nM phi29 DNA polymerase for another 10 min. After that, the rest of the components (see initiation assay above) were added. A reaction without TP was performed as a negative control. Reactions with 150 nM of wild-type TP or double-Strep-tagged wild-type TP (strep TP) were performed as a control for the total activity of these proteins. As a test of the competition, the TP mutant S232C, which shows a wild-type interaction with the DNA polymerase but is essentially inactive [27], was incubated with the wild-type TP or the strep TP. Samples were processed as described for the initiation assay described above.

#### Electrophoretic Mobility Shift Assay (EMSA)

A 216 bp DNA fragment corresponding to the *B*. *subtilis* yshC gene [Holguera, 2014 #451] was labeled at the 5’ ends by treatment with T4 polynucleotide kinase and [γ-^32^P]ATP. Non-incorporated nucleotide was removed by filtration through mini Quick Spin DNA columns (Roche). The incubation mixture contained, in 20 μl, 50 mM Tris-HCl, pH 7.5, 4% (v/v) glycerol, 1 mM DTT, 0.1 mg/ml BSA, 20 mM (NH_4_)_2_SO_4_, 1 nM of the labeled yshC gene and the indicated concentrations of TPs. The reactions were incubated for 5 min at 4°C and were analyzed in 4% polyacrylamide gels by electrophoresis in a buffer containing 12 mM Tris-acetate, pH 7.5 and 1 mM EDTA at 4°C for 1.5 h. Gels were dried and detected by autoradiography.

#### Amplification assay

The incubation mixture contained, in 25 μl, buffer MRI plus 20 mM (NH_4_)_2_SO_4_, final concentration, and 100 μM dNTPs. The concentrations of the 4 proteins of the phi29 replicative system were 6 nM DNA polymerase, 25 nM of the mutant TPs, 23 μM of p5, and 5 μM, in the case of plasmid pETORPHI, or 30 μM, in the case of phi29 TP-DNA, of p6. DNA polymerase and TPs were incubated together for 15 min before the reaction. 50 nM of TP-DNA or 170 nM of plasmid pETORPHI, linearized with DraI, were used as DNA template and the samples were incubated for 2 h at 22°C, in the case of pETORPHI, or 30°C, in the case of TP-DNA. The reactions were stopped by adding 10 mM EDTA and 0.1% SDS, final concentrations. Samples were analyzed in 0.7% (w/v) agarose gels and detected by ethidium bromide staining. Quantification of the products was carried out with the software ImageJ.

For protein i38cys, the incubation mixture contained, in 25 μl, buffer MRI plus 5 mM (NH_4_)_2_SO_4_, final concentration, and 100 μM dNTPs. The concentrations of the 4 proteins of the phi29 replicative system were 15 nM DNA polymerase, 97 nM of TP i38cys, 23 μM of p5 and 0.8 μM of p6. 170 nM of plasmid pETORPHI was used as DNA template. Samples were treated as described previously.

## Results

### Identification of permissive insertion sites on phi29 TP by transposon mutagenesis

**Fig 1:**
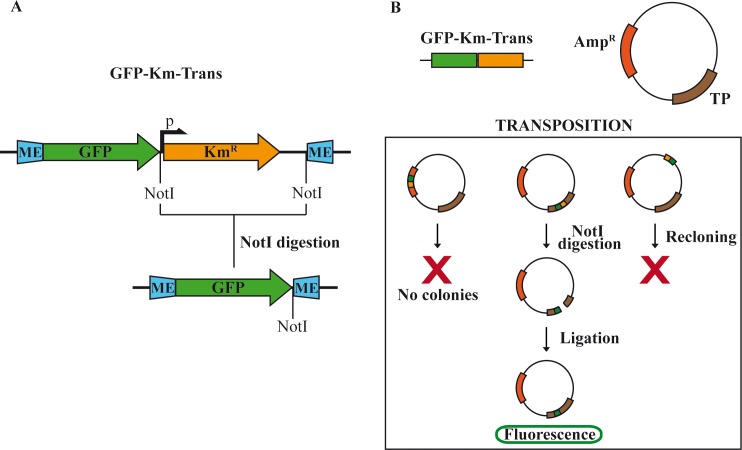
Construction of the transposition library. (A) Scheme of the donor plasmid showing the intact transposon and the inserted sequence produced after digestion with NotI and ligation. The transposon comprises two mosaic ends (ME) or transposition signals, the GFP coding sequence and a kanamycin resistance cassette (Km^R^) flanked by two NotI sites. (B) Scheme of the TP-expressing receptor plasmid and the possible outcomes after the transposition and elimination of the kanamycin cassette (see Materials and Methods for a detailed description).

In order to detect permissive insertion sites in the TP sequence we chose a Tn5-based GFP transposition strategy. The GFP was chosen because it provides an easy detection method, it has a well studied autonomous folding and its N and C ends are close to each other [28], so, presumably, the disturbance of the acceptor protein structure would not be too strong. The transposon method we used has been reported before to detect permissive insertions in other proteins [21,29] [30]. The transposon (Fig 1A) has optimized tranposition signals (mosaic end, ME, see S1 File), a GFP coding sequence devoid of transcription and translation, start or stop signals, and a kanamycin resistance gene flanked by NotI sites. As the receptor plasmid, we used the pStrepTP plasmid, a N-terminus double Strep-tag II TP expression vector based on the T7 promoter (S2 File). Once subjected to the reaction with the transposon in the presence of the Tn5 over-active transposase [31], the receptor plasmid could, in theory, acquire the transposon at any point of its sequence compatible with the plasmid replication and the antibiotic resistance function (Fig 1B).

To select the transposition events that occurred within the TP sequence we excised the DNA fragment that had a size corresponding to the TP gene plus the transposon and recloned it onto the original vector backbone. After pooling the resulting library, the kanamycin gene and adjacent sequences were eliminated by digestion with NotI and religation (Fig 1B). After that, just the GFP coding sequence, the 19 bp transposon ME ends and two short linkers remained inserted. From this procedure, we obtained a library of about 2000 clones in which the GFP coding sequence was inserted, in principle at any position in the TP gene, and in any of the two possible orientations.

To detect in-frame insertions of the GFP coding sequence into the TP gene (in theory, one out of six events of transposition within the TP gene) we induced the expression of the TP from individual clones from the final transposition library and measured the fluorescence of the cultures in the presence of appropriate controls. Out of 320 individual clones measured, we selected 56 cultures that showed a fluorescence signal clearly above the background and near that of GFP-expressing positive controls. To confirm the presence of GFP insertions on the TP protein in the selected clones, we performed Western blotting with anti-TP antibodies on extracts of the selected cultures and continued to study only those clones that showed a protein band of a size equivalent to TP plus GFP (S1 Fig, panel A, S1 Table).

**Fig 2:**
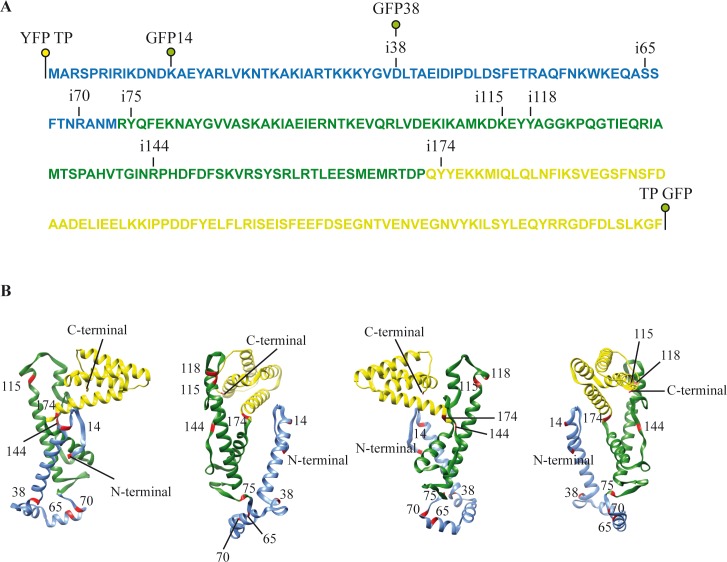
Localization of the GFP or 15 amino acids insertions on the sequence and tertiary structure of the TP. (A) The positions of YFP, GFP or 15 amino acid insertions (i) are marked on the sequence of the TP. The different domains of the TP are indicated with colors: N-terminal (blue), Intermediate (green) and C-terminal (yellow). (B) Ribbon diagrams of the 3D structure of the TP. The 3D modeling was performed with the software UCSF Chimera based on the crystallographic data from the file with the Protein Data Bank (PDB) accession number **2EX3** and on a reconstruction of the N-terminal domain using the program I-Tasser. Labels show the positions of the GFP/YFP or the 15 amino acid insertions. The color code for the different domains is the same as in A. Representations of the structure from different angles are shown for better visualization.

We selected 11 candidate insertions from the Western positive clones and carried out an assay to detect TP-specific initiation of replication of the phi29 TP-DNA on whole cell-extracts (S1 Table). We observed that, with just three exceptions (S1 Fig, panel B), none of the fusions obtained showed initiation activity. After sequencing, the three replication positive clones corresponded to the same insertion at amino acid 14 in the TP sequence (Fig 2A, GFP14). The rest of the GFP insertions fell on the Nt domain (3 insertions), on the I domain (4 insertions) and 1 insertion at the Ct domain (Fig 2A). In Fig 2B are shown the positions of the insertions on the structure of the TP, for the domains I and Ct, modelled with the software UCSF Chimera [32] and on the reconstructed structure of the Nt domain performed with the program I-Tasser [33]. We considered that the whole GFP protein could be too voluminous to allow the correct folding of the TP and be compatible with its function, and also that, for our purposes, a smaller insertion could be functional and still useful as a probe for permissive sites. So, we eliminated most of the GFP coding sequence by doing PCR with oligonucleotides (see Table 1) that hybridized divergently from the transposition signals and directed the amplification of the plasmid plus the TP gene but excluded the GFP sequence. Each primer also included an EagI site for recircularization of the plasmid. After amplification, EagI cutting and ligation, in each of the clones was left an in-frame insertion of 45 base pairs on the TP gene (S3 File). The corresponding proteins with 15 amino acids insertions were purified with Strep-Tactin columns with the results shown in S2 Fig. The protein concentrations for all the TP variants were quantified by coomasie-staining, and the TP variants were assayed for activity comparing them with the wild-type TP in order to determine a suitable insertion point. To have better standards for comparison, we also purified 2 whole GFP/YFP fusions, one is a YFP fusion at the N-terminus of the TP (YFP TP) that was already assayed *in vivo* [14] and the other is a GFP fusion at the C-terminus of the TP (TP GFP). This last protein has not been characterized before. We also included two whole GFP insertion mutants in the assays, GFP14 (insertion of the GFP at position 14) and GFP38 (insertion at position 38). GFP14 showed near wild-type activity and GFP38 showed some activity, although very low (S1 Table).

### Initiation of replication and competition with wild-type TP

**Fig 3:**
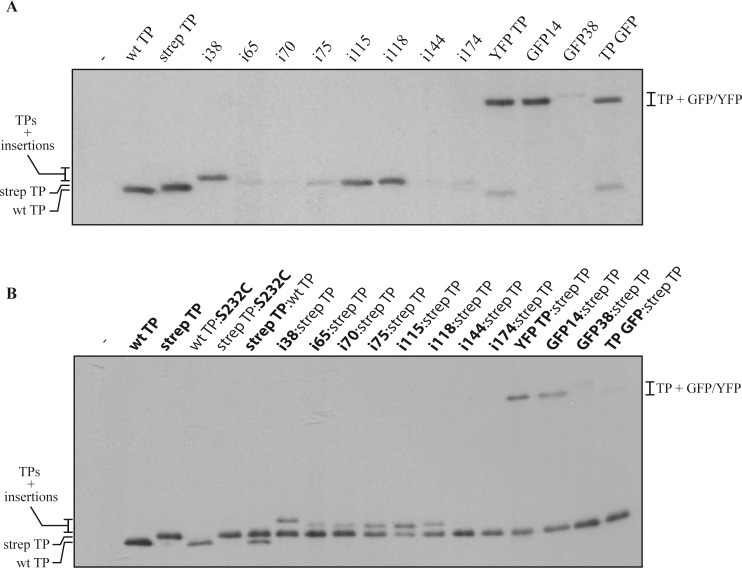
**(A) Initiation assay. TP-dAMP complex formation with wt. TP and the insertion mutants.** The initiation reaction was performed and analyzed as explained in Materials and Methods, using phi29 TP-DNA as template and [α^32^P]dATP as labeled nucleotide. After removing the excess of nucleotides, the labeled proteins were analyzed by SDS-PAGE and detected by autoradiography. The mobilities corresponding to the different TP versions are indicated. Lane -, negative control in the absence of TP; wt TP, wild-type TP; the rest of the TP variants follow the main text nomenclature. **(B) Assay of competition between TPs.** The reactions were performed and analyzed essentially as in A with the following modifications (see also Materials and Methods): 75 nM of each TP ([TP_total_] = 150 nM) were mixed and incubated in the reaction solution for 5 min prior to the addition of the DNA polymerase and then the rest of the components of the reaction. The two competing TPs are indicated as follows, strep TP:wt TP, is the competition between wt TP and the TP with double Strep-tag. The Figure is representative of at least three independent experiments. See S2 Table for quantification.

To determine if the different TP insertion mutants could behave as the wild-type TP we performed an initiation of replication assay using phi29 TP-DNA as template, the phi29 DNA polymerase, and the different TPs as primers. The results (Fig 3A) showed that the strep TP, the whole GFP fusions YFP TP, GFP14 and TP GFP, and the insertion mutants i38, i115 and i118, had activities very similar to that of the wild-type TP, while the rest, i65, i70, i75, i144, i174 and GFP38 had low levels of initiation activity (see S2 Table for quantification).

Given the fact that a difference of 15 amino acids on the TP can be resolved in standard SDS-PAGE analysis we performed competition assays under the same conditions as the previous experiment except that the wild-type equivalent strep TP and individual insertion mutants were present simultaneously in the reaction, and the initiation products were analyzed to detect the relative contributions of wild-type and mutant TPs. This allowed us to evaluate the comparative functionality of the mutants. As shown in Fig 3B, when we use an essentially inactive TP (mutant S232C, that has otherwise near wild-type interaction capabilities) as a control, there is less initiation band from the wild-type TP (lane wt TP:S232C), probably due to the competition between both proteins to bind to the DNA polymerase or replication origins. When the S232C mutant is paired with the strep TP, as expected, no band appears at the untagged TP position and only the tagged, active protein, is labeled in the initiation reaction. When the wild-type TP is paired with the strep TP, two bands appear, given that both proteins are active. Curiously, the band from the tagged protein is more intense (upper band), indicating that the double Strep-tag confers some advantage to the TP, probably in the interaction with the phi29 DNA polymerase. Then, the different mutants were paired with the strep TP and the results show that the mutants i38, i75 and i118 yield upper bands with an intensity 20–25% that of the strep TP, so this means that they can compete in the reaction but they have less capacity to support initiation that the strep TP (see S2 Table for quantification). The fusions YFP TP and GFP14 show intensities similar to the strep TP, while the mutants i65, i70, GFP38 and TP GFP produce bands of low intensity (Fig 3B). Interestingly, the mutant i115 yields a band more intense than the strep TP indicating that it has an enhanced capacity for initiation, probably due to a new set of favorable interactions with the DNA polymerase and/or the phi29 TP-DNA template. Altogether, these results indicate that most of the insertions impair the TP replication initiation function, while the fusions YFP TP and GFP14 are very similar to the wild-type TP, and the insertions i38 and i115, although less efficient than the wild-type TP, could potentially point to useful insertion sites.

### Band-shift assays

**Fig 4:**
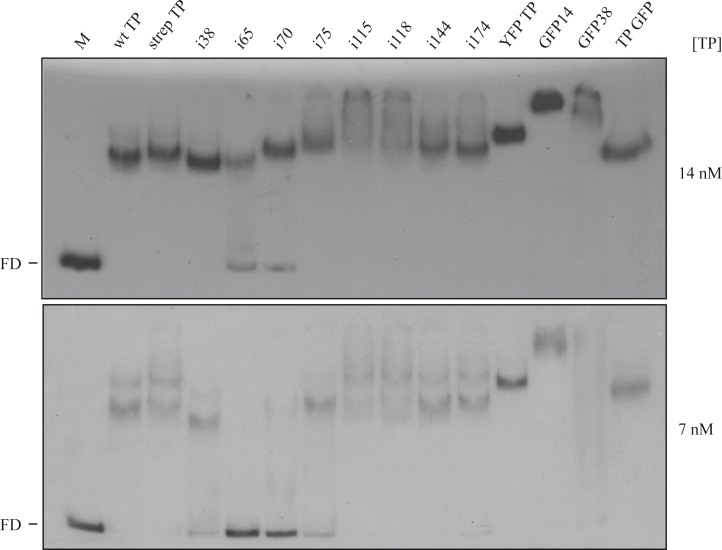
Band shift assay of the DNA binding capacity of wt TP and insertion mutants. The experiment was performed using 1 nM of a dsDNA fragment of 216 bp (corresponding to a random gene, yshC, from the *B*. *subtilis* genome [16]) labeled at the 5´ end with [γ^32^P]ATP (see Materials and Methods). The labeled DNA was incubated in the absence (lane M) or in the presence of two different concentrations of TPs (7 and 14 nM) as indicated in each panel. FD, free DNA.

Given the fact that the TP N-terminal domain has DNA binding capacity, and several of the mutants in our study have insertions within this domain or in its vicinity we checked if the mutants were affected in their DNA binding ability. We performed band-shift assays using a 219 bp dsDNA fragment as target of the purified insertion mutants. As shown in Fig 4, with 7 nM protein concentration, mutants i65 and i70 had a clearly impaired DNA binding ability while mutants i38, i75 and GFP 38 had an intermediate phenotype and the rest of the mutants and fusions were similar to the wild-type or the strep TP. At 14 nM protein all the TPs had the band shifted except i65 and i70 that still showed some DNA at the free DNA position. The results agree with the fact that disruptions on the N-terminal domain affect the DNA binding capacity of the TP, as it is the case with almost all the insertions on that domain, namely i38, i65, i70, i75 and GFP 38; however, the disruption is not too strong since it could be compensated by higher concentrations of the proteins. The GFP 14 is not affected but we have shown that a deletion encompassing those residues yields a protein active in most assays [34].

### Phi29 TP-DNA amplification with the mutant proteins

**Fig 5:**
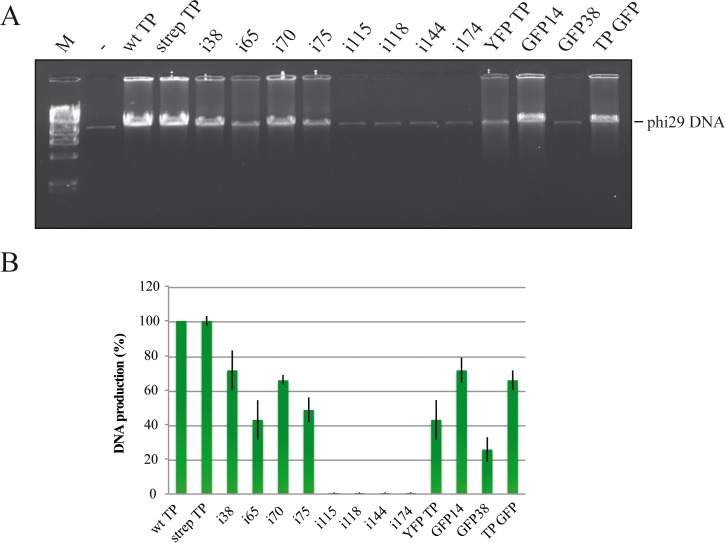
**(A) TP-DNA amplification using wt TP and the insertion mutants as primers.** The products of the amplification reactions were analyzed by agarose gel electrophoresis and ethidium bromide staining. Lane M, lambda phage DNA digested with HindIII, molecular weight marker. Lane -, negative control (DNA input), amplification reaction without TP. The band with a mobility corresponding to phi29 DNA is indicated. **(B) Bar graph with the quantification of the bands corresponding to phi29 TP-DNA.** It was calculated from the results of experiments as that shown in panel A. Production of full-length phi29 TP-DNA was calculated for each TP variant considering the band produced by the wt TP as 100%. Bars show the average and SD of three independent experiments.

Since TP-primed DNA amplification is a critical step for further applications of our system, the mutant proteins were tested in amplification experiments using phi29 TP-DNA as template, and the products were analyzed by detection on agarose gels with ethidium bromide staining (Fig 5A and 5B). Intensities of bands were standardized by setting the amount of product obtained with wild-type TP as 100%. The proteins wild-type TP and strep TP produced the same amplification, so the Strep-tag seemed not to affect the activity of the TP in amplification. Mutants i38, i70, GFP14 and TP GFP yielded amplification bands equivalent to 65–71% that of the wild-type TP. With proteins i65, i75 and YFP TP amplification values 42–48% of the wild-type TP were obtained. GFP38 protein gave a band about 25% that of wild-type TP and with the mutants i115, i118, i144 and i174 no amplification was observed. It is interesting to note that, except for i75, whose insertion is just between the Nt and I domains, the proteins that did not give amplification contain inserts located in the I or the Ct domains of the TP. Also, curiously, the mutants i65 and i70, that had shown clear defects in the initiation reaction, in the competitive initiation and in DNA binding, in this assay they produced more than 40% the amplification of the wild-type TP. The high concentration of the four dNTPs used in the amplification assay can probably explain why the defects of the i65 and i75 mutants appear relieved in this assay. On the contrary, the fusion YFP TP that behaves essentially as the wild-type TP in most assays is not as active in the amplification assay. On the other hand, we observe that the insertion of the whole GFP in the DNA binding domain, position 38, produces more disturbance on the activity than the smaller insertion (i38). The reasons for the disturbance could be folding interference, steric hindrance or both.

### TP-primed heterologous DNA amplification

**Fig 6:**
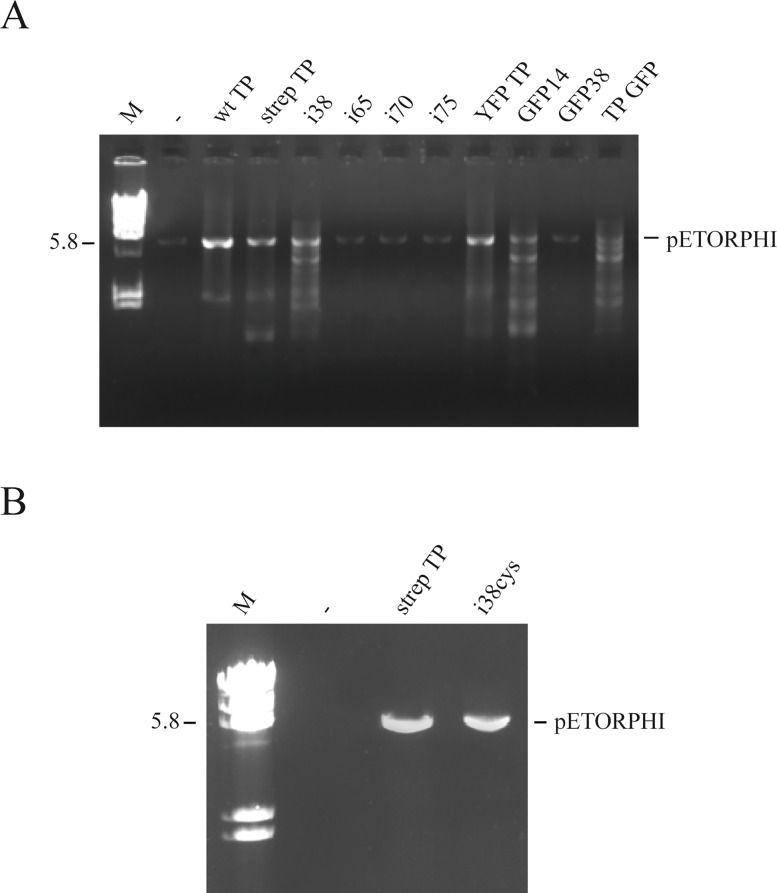
**(A) Amplification of the linearized plasmid pETORPHI using wt TP and the more active insertion mutants as primers.** The amplification reactions products were analyzed as in Fig 5. Lane M, lambda phage DNA digested with HindIII. Lane -, negative control (DNA input), amplification reaction without TP. The band with a mobility corresponding to pETORPHI is indicated. **(B) Amplification of the linearized plasmid pETORPHI using wt TP or i38cys as primers.** The template preparation and amplification reaction were performed as described in Materials and Methods. Lane M, HindIII digested lambda DNA.

As a further test of the function of the mutants, we performed TP-primed amplification using as template the linearized pETORPHI plasmid, that has the phi29 DNA ends but does not have TP covalently linked at the ends; this assay is also the closest test for potential applications of the system, and the TPs that work in this test should be the candidates for further study. We consider this test the best because it allows to amplify any DNA that has the 200bp ends from the Phi29 genome. A series of experiments were performed under different conditions, and the experiment shown in Fig 6A was done using the conditions that allowed the highest activity of the mutants (see Materials and Methods). Also, due to the absence of activity in previous assays, mutants i115, i118, i144, i174 were not included in this experiment. The amplification obtained using the strep TP was somewhat lower than the amplification produced by the wild-type TP, both compared with the input (Lane -). It is also observed that the only mutants and fusions that supported amplification were, i38, YFP TP, GFP 14, and TP GFP, although in all cases, several bands with lower molecular size than the unit length (5,834 bp, linearized pETORPHI) were obtained. On the other hand, the insertion mutants i65, i70, i75 and GFP 38 did not yield detectable amplification bands. The fusion YFP TP was the one that performed the best in this assay in terms of the intensity of the band and the low amount of non-wanted products, however, it is not an insertion as such, and it was not the primary focus of the screening. The fusion TP GFP produces amplification of the target plasmid but the amount of non-unit length bands is high. Mutant i38 was the second best in this assay; it produces a reasonable amount of unit-length amplified DNA and performs at near wild-type levels in other assays. Therefore, we selected position 38 as the most permissive position for insertions on the TP sequence and used it for further study. Also, the insertion at position 38 is interesting because it separates the DNA binding domain and NLS sequence (amino acid 38 to the amino end) from the rest of the protein that could still be linked to the DNA (39 to the C-end). Mutant GFP14 is much less interesting because it gives more non-wanted amplification products and also, importantly, it does not separate the DNA binding domain from the rest of the TP.

### Insertion substitution on i38 TP

The DNA binding capacity of the TP could be a problem in implementing a system for gene transfer since it could interfere with different processes such as transport or recombination. The naturally present NLS, on the other hand, is useful to target the DNA to the nucleus, however one may want the DNA construct to remain in the cytoplasm or to be transported to other compartments. If we can eliminate the DNA binding domain/NLS after the amplification reaction and then add a different peptide module to the rest of the TP sequence, then we would have a very flexible vehicle to test on DNA delivery. So, we changed the 15 amino acids sequence of the i38 mutant and introduced a protease site that would allow to eliminate the segment from the insertion to the N-terminus plus a short glycine linker that contains also a cysteine. The cysteine, that is not present in the wild-type TP, would allow the conjugation to peptidic modules carrying functions reactive to sulfhydryl groups. The inserted sequence was (GGLVPRGSGGCGG) to replace the original insertion in i38. The new TP insertion mutant (i38cys) was constructed and purified, and the protein was tested under different conditions to optimize the pETORPHI amplification assay. In Fig 6B we show that the amplification obtained with the mutant i38cys after optimization of the reaction conditions (5 mM (NH_4_)_2_SO_4_, 97 nM of TPi38cys, 0.8 μM of p6 and 170 nM of plasmid pETORPHI, rest of the conditions in Materials and Methods) is comparable to that of the strep TP.

## Discussion

Transposon-based linker scanning mutagenesis has been used to probe the TP sequence for permissive insertion points. In the first round of mutagenesis the whole GFP coding sequence was inserted in a series of positions along the TP, resulting, with only one exception, GFP 14, in TPs devoid of activity as measured by the initiation of replication assay. While the GFP has been inserted in some proteins without loss of activity [35, 36], it is not surprising that the interruption of the folding of a target protein, to allow the GFP to fold itself, and then the resumption of the target folding, can easily thwart the formation of the correct structure of the target protein [37] or, alternatively, preclude its function by steric hindrance. Therefore, we proceeded to eliminate most of the transposed sequence from selected clones, leaving insertions of 15 amino acids as the test for permissive sites. In this way, we recovered at least some of the activity in most of the clones. Here, the best example is the position 38 of the TP that is rendered almost non-functional upon the insertion of GFP, however, is nearly wild-type when the insertion is reduced to 15 amino acids. Apart from position 38 only the fusions at the N-terminus, position 14 and C-terminus keep most of the activity of the wild-type TP in all assays, with all these positions having the whole GFP as insertion. In general, fusions at the N or C-terminus of proteins are well tolerated, although, for the TP, the behavior of the C-terminal fusion shows that this position is less permissive than the N-terminus. In previous work, it has been shown that the first 14 amino acids of the TP are dispensable for function, while, on the other hand, by removal of 3 amino acids from the C-end the initiation activity is reduced up to 50% [13]. This helps to explain the higher activities of the N and the 14 fusions in all assays and the behavior of the C-terminal fusion.

Regarding the results obtained with the other insertions, they show good agreement with what is known about the domains of the TP. So, insertions at the N-terminus (i38, i65, i70, and i75, which is immediate to the Nt domain) affect the DNA binding capacity of the TP, but do not eliminate the rest of the activities. Insertions on the I domain (i115 and i118) show near wild-type initiation but totally fail to support amplification, underscoring the role of the I domain in the transition process. Interestingly, the i115 mutant has an enhanced ability to compete the wild-type TP in initiation, probably due to enhanced interactions, via the inserted sequence, with the DNA polymerase. This could be the basis to design a specific inhibitor of the phi29 DNA polymerase or an affinity reagent. Finally, mutants i144 (insertion at the I domain) and i174 (insertion at the Ct domain) are essentially inactive in all assays except in DNA binding. This phenotype is typical of mutants at the Ct domain or regions that are essential for the initiation stage, that involves a precise binding and interaction of TP and DNA polymerase.

**Fig 7:**
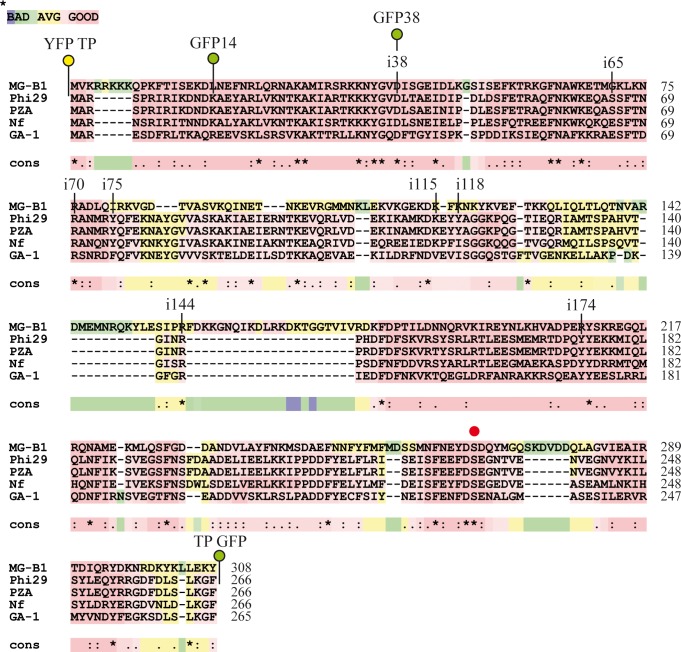
Alignment of the protein sequences of the TPs of bacteriophages MG-B1, phi29, PZA, Nf and GA-1. The alignment was performed with the software T-Coffee. The alignment shows a large insertion at the equivalent of position 144 of the phi29 TP. The serine involved in the phosphoesther bond is marked with a red dot. The color code indicates the reliability of the alignment for each sequence, from red (good) to blue (poor). In the lower bar there is an evaluation of conservation for each residue, (*) conserved, (:) conservative change, (.) semiconservative change or () non-conserved.

The TP interacts with the DNA, as seen in the crystal, and it has also large contact surfaces with the DNA polymerase. Of the many point mutants of the TP that have been characterized up to date, most show impaired activity respect to the wild-type TP [38–41]. This fact suggests that the TP protein sequence has very few insertion points that are compatible with a high level of function, and this could be an explanation as why we have not found more permissive insertion sites. A phage genome recently added to the databases, MG-B1 [42], encodes a protein with high similarity to the phi29 TP (Fig 7). Interestingly, this putative TP has a number of insertions with respect to the phi29 TP and the largest insertion (36 amino acids) occurs at the equivalent of position 144 of the phi29 TP, as can be observed in the alignment, performed with the T-Coffe software [43]. So, taking into account that our screening has detected eight structurally permitted insertions, and the fact that one of the insertions coincides with the position of the largest insertion of the only reported phi29-like TP that has sufficiently large insertions, we believe that the representativity of our screening is adequate. Also, in our screening, some of the mutants were isolated several times independently what suggests that the coverage we have reached is good.

The insertion i38 is of particular interest from the point of view of adding new functions or features to the phi29 TP, towards the goal of engineering a gene delivery vehicle. As mentioned in the Introduction, residues 1 to 38 of the TP are necessary for DNA binding activity, to localize in the bacterial nucleoid, and they are the minimal *bona fide* NLS present in the TP. A protease site such as we have placed at position 38 can eliminate the NLS/DNA binding ability from the TP. Given that the protease site is within a flexible linker the efficiency of cutting would tend to be optimized. The introduction of a cysteine also at this linker, taking into account that there is no other cysteine in the TP sequence, gives an anchoring point for conjugatable peptides that would carry desired functions such as cell-penetrating peptides. As we have shown, 15 and 17-residue insertions at position 38 yield active proteins in the TP-primed amplification of a linearized plasmid, so, most probably, other insertions would be also tolerated. The utility of the protease site and the cysteine combined can result in a versatile TP-platform to develop a new gene delivery system with improved properties, and the insertion point could be also used in other possible engineering schemes on the TP.

## Supporting Information

S1 Fig **(A) Western analysis to detect fusions TP-GFP expressed by selected clones.** The putative presence of TP-GFP fusions was detected by western blotting using anti-TP antibodies. The lanes corresponding to relevant TP derivatives are labeled. Lanes X, non-relevant clones. **(B) TP-dAMP formation assay using extracts from bacterial clones expressing TP derivatives.** The assay was performed as described in Materials and Methods. Lane pT7, negative control, clone harbouring pT7-3; lane YFP-TP, positive control, clone harbouring plasmid pYFP-TP, expressing a N-terminal fusion of YFP to the TP; Lanes X, non-relevant clones. The electrophoretic mobilities of TP and YFP-TP are indicated.
(TIF)

S2 Fig Purification of selected TP insertion mutants. The proteins were purified using a double Strep-tag II and Strep-Tactin columns as described in Materials and Methods and were analyzed by SDS-PAGE and Coomassie blue stainning. A molecular weight marker with bands corresponding to 36, 42 and 54 KDa was included (lane M).
(TIF)

S1 File Sequence of transposon mTn5[GFP-Nla1]. Mosaic ends are indicated in italics, GFP coding sequence underlined, NotI sites in bold.
(DOCX)

S2 File pStrepTP plasmid. Underlined double Strep-tag. Uppercase TP coding sequence.
(DOCX)

S3 File Designed inserted sequence. (DOCX)

S1 Table Comparison of the different activities of the selected clones. * Fluorescence data expressed in terms of the percentage relative to the average of the values of the fluorescence of a TP linked to YFP.
(TIF)

S2 Table Quantification of the initiation assays and the assays of competition between TPs. Values are average and SD from at least three independent experiments. All values are normalized to the activity of the wild-type TP, set as 1.00, for each experiment. In the competition experiment the experimental points wt TP, strep TP, wt TP:S232C and strep TP:S232C correspont to the intensity of the only band present in the lanes. For the rest of the points, as an example, in the i38:strep TP line, the first column corresponds to the intensity of the i38 band while the second column corresponds to the band of the strep TP in the same lane. The strep TP is present in competition with all the TP variants from the fifth lane on. The third and fourth columns are the SDs of the first and second columns respectively.
(TIF)
