# Supplementary figures and images for: Engineering Permissive Insertion Sites in the Bacteriophage Phi29 DNA-Linked Terminal Protein
Source: PLoS One. 2016 Oct 25;11(10):e0164901. doi: 10.1371/journal.pone.0164901 (PMC5079584; doi:10.1371/journal.pone.0164901)

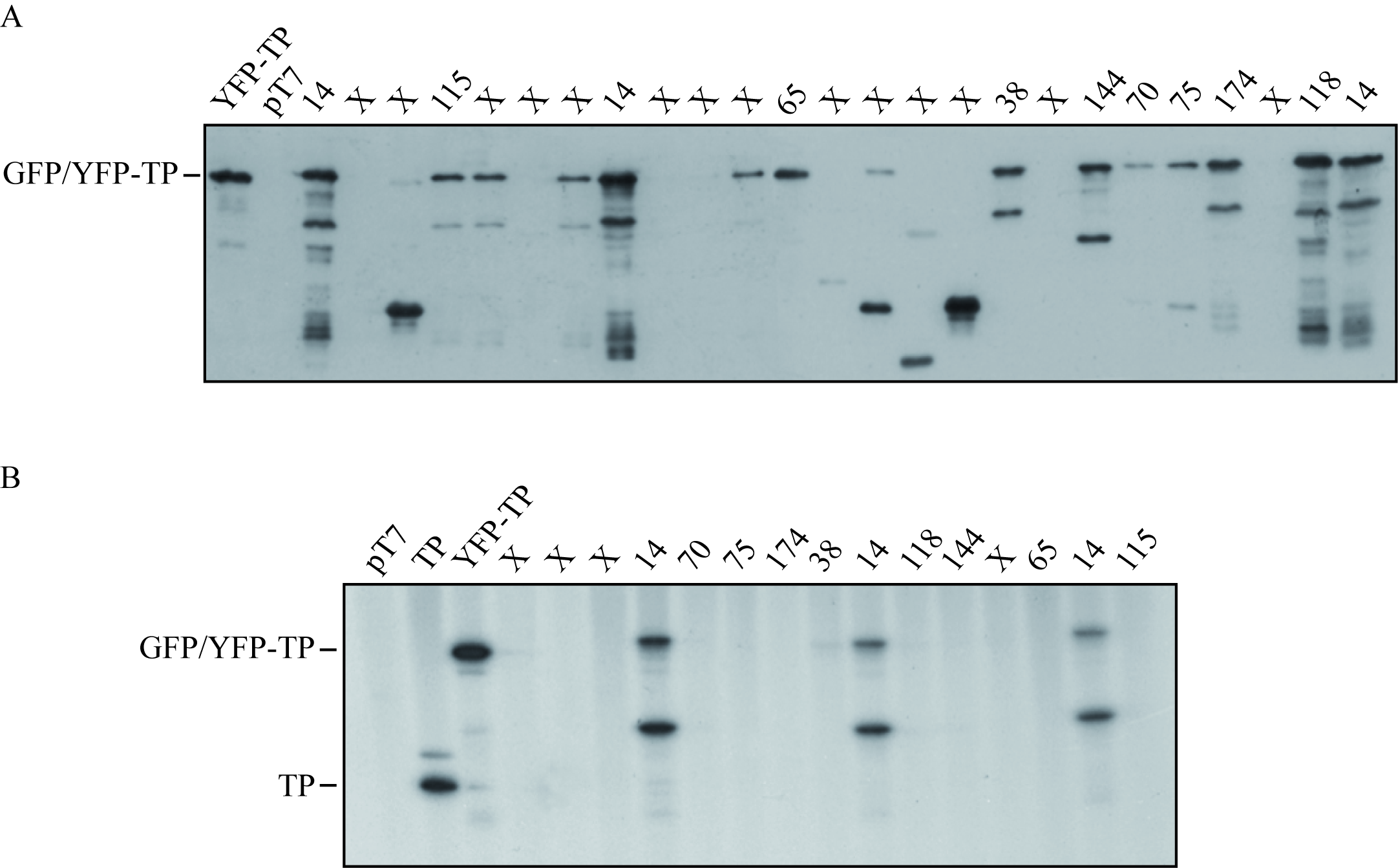

Supplement: S1 Fig — (A) Western analysis to detect fusions TP-GFP expressed by selected clones. The putative presence of TP-GFP fusions was detected by western blotting using anti-TP antibodies. The lanes corresponding to relevant TP derivatives are labeled. Lanes X, non-relevant clones. (B) TP-dAMP formation assay using extracts from bacterial clones expressing TP derivatives. The assay was performed as described in Materials and Methods. Lane pT7, negative control, clone harbouring pT7-3; lane YFP-TP, positive control, clone harbouring plasmid pYFP-TP, expressing a N-terminal fusion of YFP to the TP; Lanes X, non-relevant clones. The electrophoretic mobilities of TP and YFP-TP are indicated. (TIF) [file pone.0164901.s001.tif]

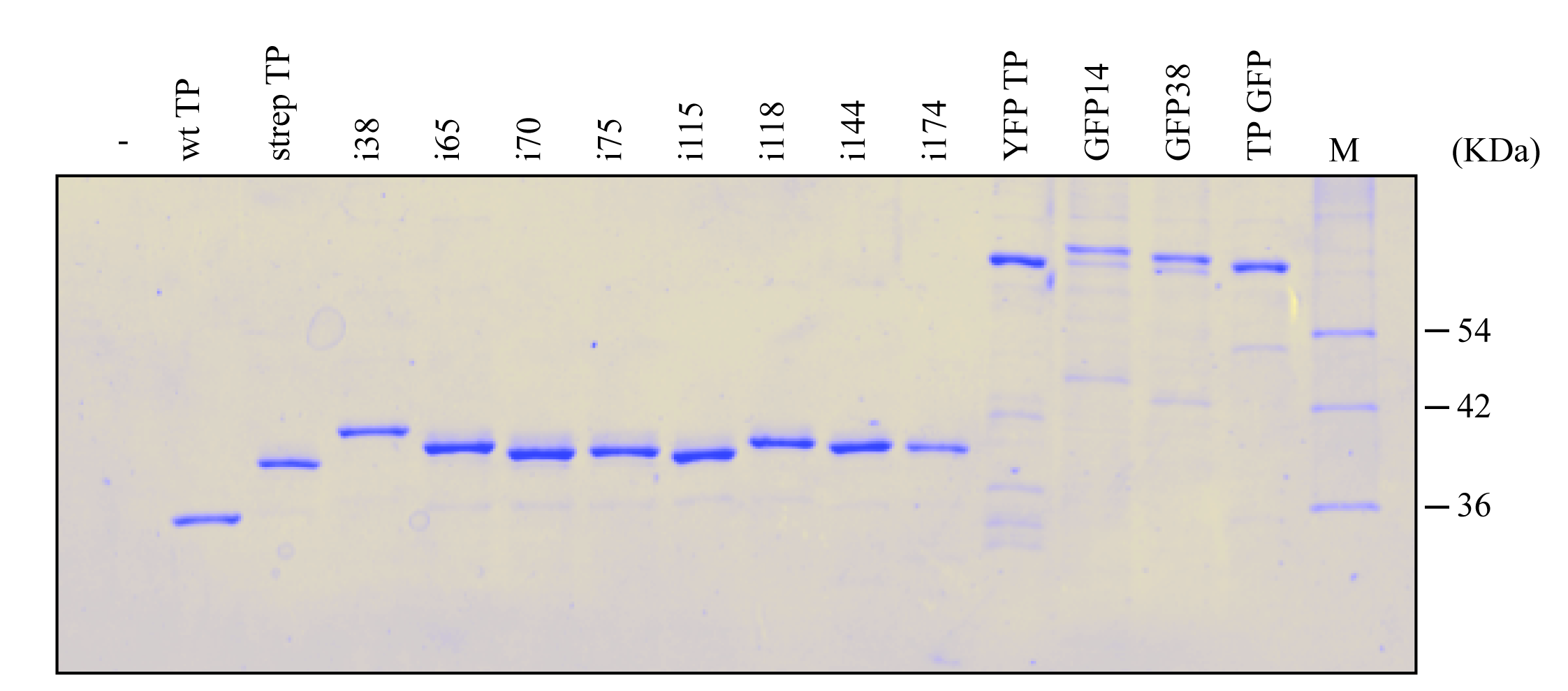

Supplement: S2 Fig — The proteins were purified using a double Strep-tag II and Strep-Tactin columns as described in Materials and Methods and were analyzed by SDS-PAGE and Coomassie blue stainning. A molecular weight marker with bands corresponding to 36, 42 and 54 KDa was included (lane M). (TIF) [file pone.0164901.s002.tif]

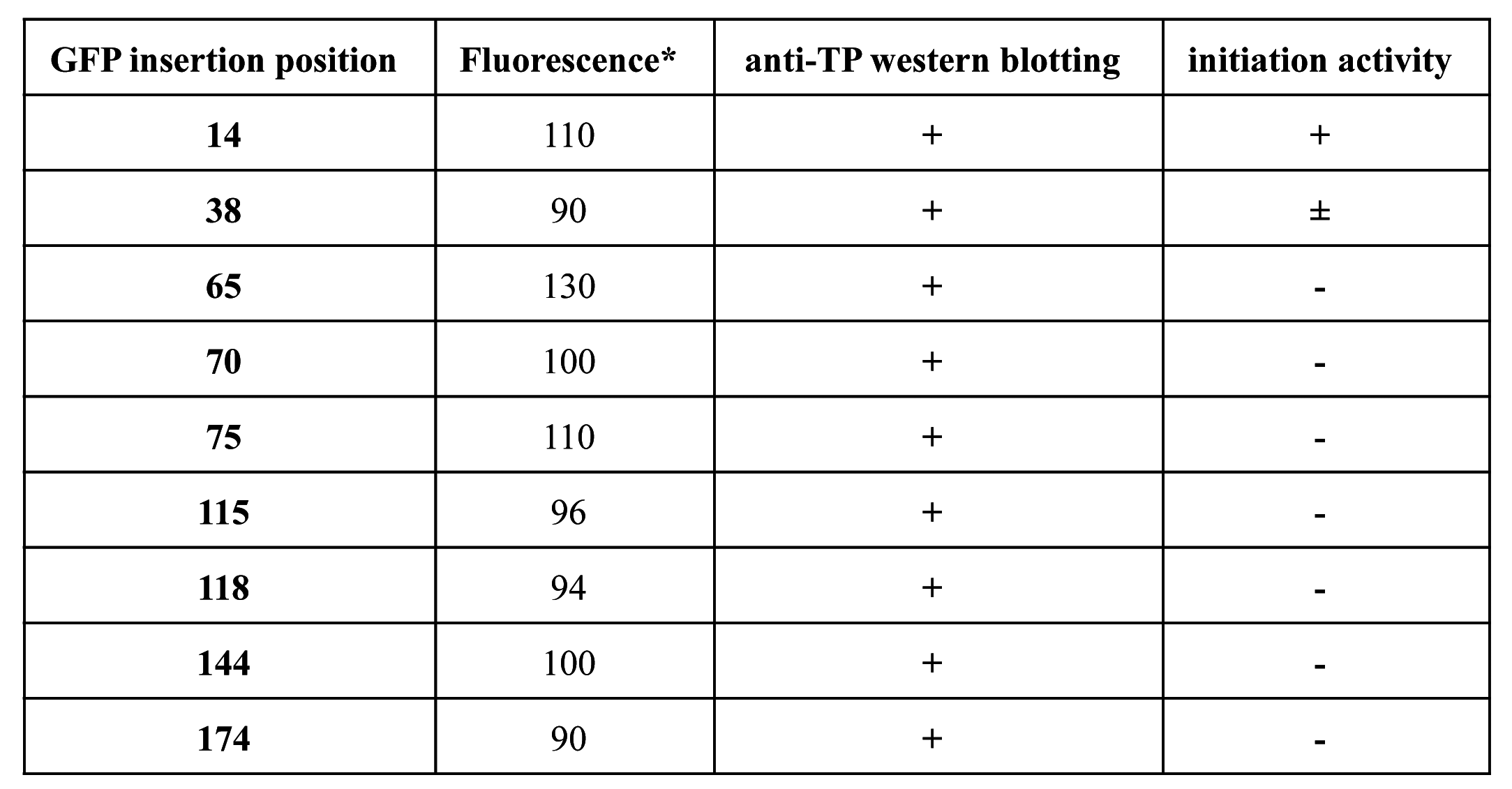

Supplement: S1 Table — * Fluorescence data expressed in terms of the percentage relative to the average of the values of the fluorescence of a TP linked to YFP. (TIF) [file pone.0164901.s006.tif]

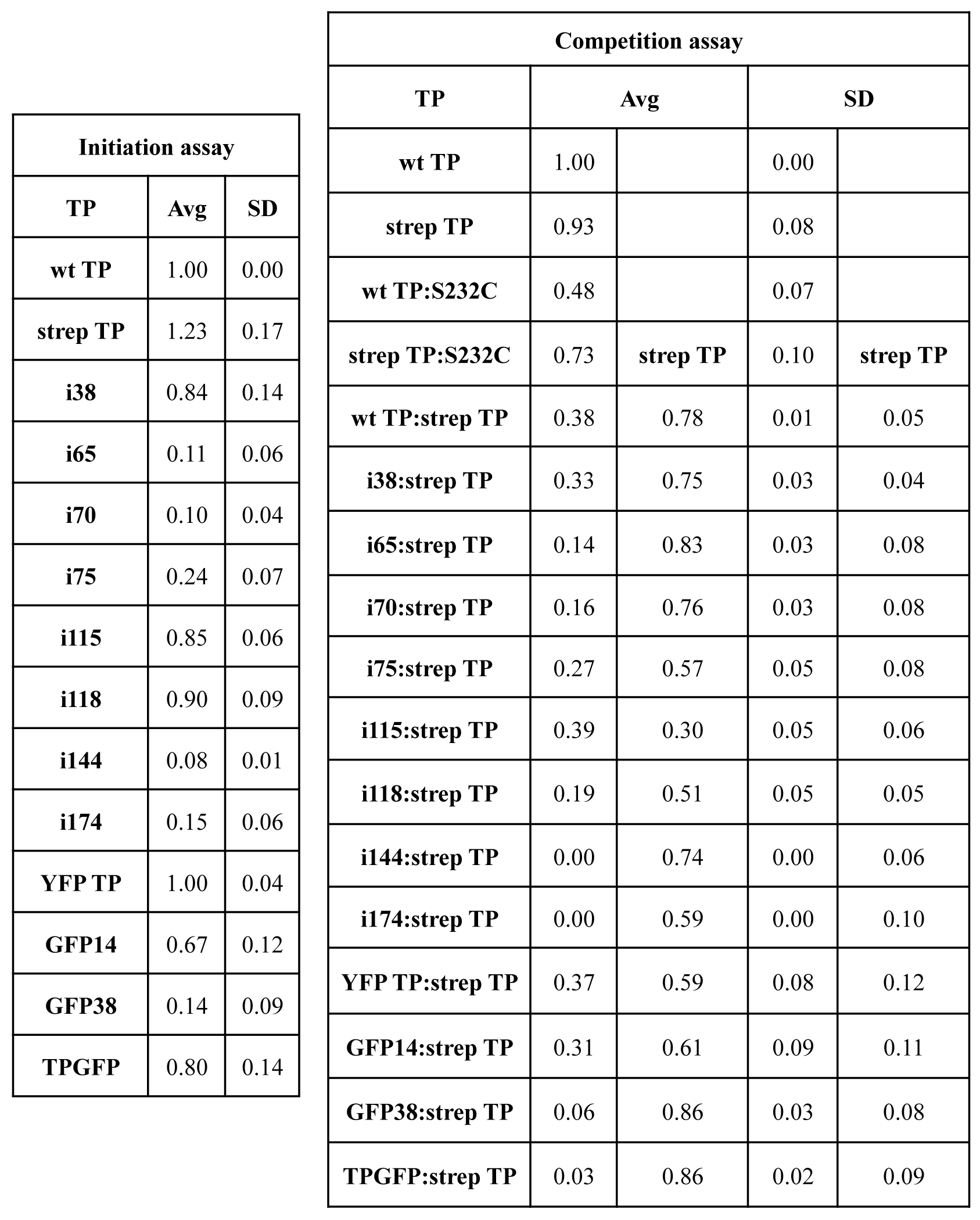

Supplement: S2 Table — Values are average and SD from at least three independent experiments. All values are normalized to the activity of the wild-type TP, set as 1.00, for each experiment. In the competition experiment the experimental points wt TP, strep TP, wt TP:S232C and strep TP:S232C correspont to the intensity of the only band present in the lanes. For the rest of the points, as an example, in the i38:strep TP line, the first column corresponds to the intensity of the i38 band while the second column corresponds to the band of the strep TP in the same lane. The strep TP is present in competition with all the TP variants from the fifth lane on. The third and fourth columns are the SDs of the first and second columns respectively. (TIF) [file pone.0164901.s007.tif]
