# Supplementary material for: Engineering Permissive Insertion Sites in the Bacteriophage Phi29 DNA-Linked Terminal Protein
Source: PLoS One. 2016 Oct 25;11(10):e0164901. doi: 10.1371/journal.pone.0164901 (PMC5079584; doi:10.1371/journal.pone.0164901)
Supplement: S1 File — Mosaic ends are indicated in italics, GFP coding sequence underlined, NotI sites in bold. (DOCX) [file pone.0164901.s003.docx]

**S1 File. Sequence of transposon mTn5[GFP-Nla1]**

Mosaic ends are indicated in italics, GFP coding sequence underlined, NotI sites in bold.

*CTGTCTCTTATACACATCT*CCTGCAGGAGTAAAGGAGAAGAACTTTTCACTGGAGTTGTCCCAATTCTTGTTGAATTAGATGGTGATGTTAATGGGCACAAATTTTCTGTCAGTGGAGAGGGTGAAGGTGATGCAACATACGGAAAACTTACCCTTAAATTTATTTGCACTACTGGAAAACTACCTGTTCCATGGCCAACACTTGTCACTACTTTGACTTATGGTGTTCAATGCTTTTCAAGATACCCAGATCATATGAAACGGCATGACTTTTTCAAGAGTGCCATGCCCGAAGGTTATGTACAGGAAAGAACTATATTTTTCAAAGATGACGGGAACTACAAGACACGTGCTGAAGTCAAGTTTGAAGGTGATACCCTTGTTAATAGAATCGAGTTAAAAGGTATTGATTTTAAAGAAGATGGAAACATTCTTGGACACAAATTGGAATACAACTATAACTCACACAATGTATACATCATGGCAGACAAACAAAAGAATGGAATCAAAGTTAACTTCAAAATTAGACACAACATTGAAGATGGAAGCGTTCAACTAGCAGACCATTATCAACAAAATACTCCAATTGGCGATGGCCCTGTCCTTTTACCAGACAACCATTACCTGTCCACACAATCTGCCCTTTCGAAAGATCCCAACGAAAAGAGAGACCACATGGTCCTTCTTGAGTTTGTAACCGCTGCTGGGATTACACATGGCATGGATGAACTATACAAA**GCGGCCGC**ATTTAAATTTGTGTCTCAAAATCTCTGATGTTACATTGCACAAGATAAAAATATATCATCATGAACAATAAAACTGTCTGCTTACATAAACAGTAATACAAGGGGTGTTATGAGCCATATTCAGCGTGAAACGAGCTGTAGCCGTCCGCGTCTGAACAGCAACATGGATGCGGATCTGTATGGCTATAAATGGGCGCGTGATAACGTGGGTCAGAGCGGCGCGACCATTTATCGTCTGTATGGCAAACCGGATGCGCCGGAACTGTTTCTGAAACATGGCAAAGGCAGCGTGGCGAACGATGTGACCGATGAAATGGTGCGTCTGAACTGGCTGACCGAATTTATGCCGCTGCCGACCATTAAACATTTTATTCGCACCCCGGATGATGCGTGGCTGCTGACCACCGCGATTCCGGGCAAAACCGCGTTTCAGGTGCTGGAAGAATATCCGGATAGCGGCGAAAACATTGTGGATGCGCTGGCCGTGTTTCTGCGTCGTCTGCATAGCATTCCGGTGTGCAACTGCCCGTTTAACAGCGATCGTGTGTTTCGTCTGGCCCAGGCGCAGAGCCGTATGAACAACGGCCTGGTGGATGCGAGCGATTTTGATGATGAACGTAACGGCTGGCCGGTGGAACAGGTGTGGAAAGAAATGCATAAACTGCTGCCGTTTAGCCCGGATAGCGTGGTGACCCACGGCGATTTTAGCCTGGATAACCTGATTTTCGATGAAGGCAAACTGATTGGCTGCATTGATGTGGGCCGTGTGGGCATTGCGGATCGTTATCAGGATCTGGCCATTCTGTGGAACTGCCTGGGCGAATTTAGCCCGAGCCTGCAAAAACGTCTGTTTCAGAAATATGGCATTGATAATCCGGATATGAACAAACTGCAATTTCATCTGATGCTGGATGAATTTTTCTAATAATTAATTGGACCGCGGTCCGCGCGGGTCCTGCAGGGGTGAAAGCAACGTGGTGGTGCATCAGGCGGATGAACGC**GCGGCCGC***AGATGTGTATAAGAGACAG*
