# Supplementary material for: Engineering Permissive Insertion Sites in the Bacteriophage Phi29 DNA-Linked Terminal Protein
Source: PLoS One. 2016 Oct 25;11(10):e0164901. doi: 10.1371/journal.pone.0164901 (PMC5079584; doi:10.1371/journal.pone.0164901)
Supplement: S3 File — (DOCX) [file pone.0164901.s005.docx]

**S3 File**

DNA: ctgtctcttacggccgcagatgtgtataagagacagggtgtagac

Amino acids: LSLTAADVYKRQGVD
